# The influence of linkages, feedback mechanisms, and caregiver mobility on immunization follow-up visits in Lideta sub-city of Addis Ababa, Ethiopia: a qualitative study

**DOI:** 10.1186/s12961-021-00690-5

**Published:** 2021-08-11

**Authors:** Thewodros Zewde, Alula Teklu, Diriba Bedada, Yoseph Tsehaye

**Affiliations:** 1 MERQ Consulting P.L.C, Addis Ababa, Ethiopia; 2 Lideta sub-city Health Bureau, Addis Ababa, Ethiopia

**Keywords:** Linkages, Feedback mechanism, Follow-up, Transferal process, Mobility, Transferred out

## Abstract

**Background:**

Losing children to follow-up is one of the major barriers identified in Ethiopia’s immunization programme. In many urban slum areas like Lideta sub-city, Addis Ababa, several demand- and supply-side factors affect the follow-up visits for routine immunization services, so this study aimed to explore the influence of linkages, a feedback mechanism, and caregiver mobility on immunization follow-up visits.

**Methods:**

The study team employed a qualitative method and conducted 30 in-depth interviews with caregivers, 26 interviews with key informants, and five focus group discussions with health officials and decision-makers. A deductive content and thematic analysis was carried out by importing the transcripts into OpenCodes, applying the a priori codes, and identifying new codes and themes.

**Results:**

The linkages among health facilities included those from hospitals to health centres, from hospitals to hospitals, and from health centres to health centres within and outside the sub-city. Using these linkages, most vaccinators transfer caregivers without providing multi-dose vial (MDV) vaccines, mainly bacille Calmette–Guérin (BCG) and measles-containing vaccines (MCV), “to minimize wastage” and thus successfully reduce vaccine wastage rates; yet most caregivers wasted their time, energy, and money travelling from one health facility to another. Despite some efforts to transfer caregivers using “transferal slips” and informal phone calls to vaccinators’ friends, unfortunately, there was no formally established system for obtaining feedback about the arrival of caregivers and continuation of the follow-up visits. Overall, the transfer process lacked uniformity, used various approaches, and was not systematic.

**Conclusions:**

Transferal of caregivers for the sake of minimizing wastage of MDV vaccines without checking the vaccination schedules of the receiving health facilities, using various informal types of tools and approaches, along with a poor follow-up and feedback system, were major identified challenges which cost caregivers extra money, energy, and time in getting timely immunization services. Therefore, the Federal Ministry of Health should strengthen the linkages among facilities, ensure the establishment of formal communications by developing guidelines and standardized tools – transferal slips and approaches – and initiate a fast feedback provision system using SMS text messages.

## Background

In Africa, more than 30 million children under the age of 5 suffer from vaccine-preventable diseases (VPDs) every year. Of these, over half a million children die from VPDs annually due to limited access to immunization services, accounting for 58% of all global deaths among under-5 children due to VPDs [1]. In Ethiopia, about 3% of children under 5 die due to measles [2]. Thus, almost every public health facility in the country provides 12 antigens, including the measles-rubella vaccine (MRV) and others, such as the bacille Calmette–Guérin (BCG) vaccine, oral polio vaccine (OPV), pentavalent vaccine (diphtheria, pertussis, tetanus, hepatitis B, and *Haemophilus influenzae* B), pneumococcal conjugate vaccine (PCV), inactivated poliovirus vaccine (IPV), and rotavirus vaccine (RV), through the routine immunization (RI) programmes targeting measles and the other major VPDs during childhood [3].

Concurrently, the country has a well-structured public health care system around the concept of a “health network model”, which is composed of three tiers of vertically linked levels – primary, secondary, and tertiary – each with defined catchment populations. The primary level in rural settings includes primary health care units (PHCUs), defined as five satellite health posts with a health centre and primary/district hospital. In the urban setting, the health centre is the primary entry point to the health system. Secondary-level health care includes general hospitals (zonal/regional), and tertiary-level health care includes tertiary hospitals (central/referral) [4–6]. Under vaccination service provision, demand for vaccines and vaccination is a complex concept that is not external to supply systems but rather encompasses the interaction between human behaviours and system structure and dynamics [7]. Correspondingly, the country reaches targeted groups through the existing system, and “transferal” of children for vaccination (defined in this study as “informing a caregiver of a child to go to any level of health facility to get vaccines for her child”) is common among health facilities. A child can be transferred across levels of facilities, for example from a higher-level hospital to a lower-level hospital or health centre (i.e., a vertical transfer). However, a child can also be transferred from one health centre to another of the same level (i.e., horizontally).

Additionally, there are various factors that can influence the service provision and transferal process within the structure, and in many urban slum areas, where housing and living conditions are exceptionally poor [8], one of the major problems for access to and utilization of services is losing children before follow-up visits. This means that once some caregivers have accessed and received routine immunization services for their children from one health facility at a given time, they fail to show up for their subsequent visits at the same facility based on their appointment date. On top of that, improvement of urban immunization programmes requires collaboration among health facilities [9]; the lack of well-functioning linkages and strong feedback mechanisms between private and public health facilities and among public health facilities, the absence of a formal communication system among district health offices, and the increased number of self-referrals for many reasons are some of the other causes of losing children to follow-up.

Despite the existence of various demand- and supply-side factors that may influence immunization service uptake, little is known about how these factors are playing out in urban slum areas in Ethiopia. Hence, this study explores how the functionality of linkages and feedback mechanisms among health facilities and the mobility of caregivers affect follow-up visits in routine immunization services. It assesses the steps and situations that the transferred-out caregivers encounter in the process of transferring from one health facility to another and examines the existence of functional linkages among health facilities and the availability of a feedback system, active collaboration and formalized communication between health facilities, the availability of formal and expanded programme of immunization (EPI)-specific procedures and tools for linking caregivers between health facilities, the quality of the accountability for providers’ performance and supportive supervision to improve the linkage system, and how the government can provide support for these linkages.

Subsequently, recommendations are made regarding feasible and innovative strategies to improve bilateral communications and active collaboration among health facilities that provide routine immunization services benefiting targeted children so caregivers can access these services easily and thereby reduce the number of patients lost to follow-up.

## Methods

### Study setting

The study was conducted in Lideta sub-city, Addis Ababa, Ethiopia, which was purposely selected, as it has several slum areas; poor vaccination coverage of 31.5% BCG, 44.7% Penta-1, 47.6% Penta-3, and 42.7% measles-containing vaccine (MCV); and high loss to follow-up due to construction of condominium apartments and displacement of residents. The sub-city has an area of 918.4 km^2^, 10 districts, 60 196 households, a total population of 246 803, six hospitals, six health centres, and 52 private clinics. All the health centres and four of the hospitals provide routine vaccination services.

### Study design

The study team employed a qualitative research approach to answer the main research question: how do linkages, feedback mechanisms, and caregiver mobility influence immunization follow-up visits?

### Sampling

The first 30 caregivers were purposively selected based on convenience considering their availability for follow-up. The 26 key informants and five focus group discussants were purposively selected from all levels considering their roles and contributions in the EPI programme. Except for three key informants, the rest participated in the focus group discussions (FGDs) (Table 1).

**Table 1:** Disaggregation of participants by sex and methods

Methods	Respondents	Male	Female	Total
IDIs	Caregivers	0	30	30
KIIs	UHEWs	0	3	3
	Medical directors (HCs)	4	1	5
	EPI focal (HCs + hospitals)	5	7	12
	Coordinators/supervisors (district)	3	1	4
	Coordinators/supervisors (sub-city)	1	1	2
	Total	13	13	26
FGDs	Medical directors (HCs)	6	0	6
	EPI focal (HCs)	0	7	7
	UHEWs	0	6	6
	EPI focal (hospitals)	0	6	6
	Coordinators/supervisors (districts + sub-city)	4	4	8
	Total	10	23	33

*IDI* in-depth interview, *KII* key informant interview, *FGD* focus group discussion, *UHEW* urban health extension worker, *HC* health centre, *EPI* expanded programme of immunization

### Data collection

The data collection and transcription activities took a total of 4 months (August–November 2017). In-depth interviews (IDIs) were conducted at health facilities, while key informant interviews (KIIs) were done at workplaces and FGDs at meeting halls. All contacted participants agreed to participate; hence, all interviews and discussions were conducted in Amharic using pretested guides in quiet places at a convenient time. Master of public health (MPH) degree-holder moderators and note-takers carried out the IDIs and KIIs, and the authors led the FGDs. The note-takers recorded the data using digital tape recorders and notebooks. On average, the IDIs were finished within 45 minutes, while the KIIs and FGDs took 90 minutes each. The study team ensured data saturation by listening to and discussing tape-recorded data on a weekly basis, repeating 5% of IDIs and KIIs, and returning transcripts to key informants and discussants for further validation and correction.

### Data analysis

A deductive content and thematic analysis approach was applied. First, an initial set of codes were generated and aligned with the research questions by the authors. As the initial sets of codes consisted only of major categories, the code categories were further refined, and sub-codes were developed. Then, the analysis took place by importing the transcripts into OpenCodes (QDR: https://doi.org/10.5064/F6HMZBEL) and applying the a priori codes and new codes.

### Ethical considerations

The World Health Organization Ethics Review Committee (WHO ERC) and local institutional review board (IRB) approved the protocol. No subjects were interviewed without having providing informed consent.

## Results

The findings are presented in three broad sections: existing linkages, transferal processes and caregiver mobility, and feedback mechanisms with major barriers.

### Existing linkages

The linkages among health facilities included those from hospitals (government and private) to health centres, from hospitals to hospitals, and from health centres to health centres. The linkages focused on what exists within health facilities found within or outside of the sub-city. These facilities built their linkages with facilities that were nearby and easily accessible. Within a health facility, the first linkages were initiated between the delivery service unit and the EPI unit, as newborn babies required the first vaccine (BCG) at birth. In linking caregivers from the community level with health centres, urban health extension workers (UHEWs) were the key players, with the responsibilities of identifying and transferring those children who had not attended vaccination at all or those children who had defaulted from their vaccination schedules.

### Transferal processes and caregiver mobility

#### Multi-dose vial policy (MDVP) for BCG versus inconvenient BCG vaccination sessions at health facilities

Even though most vaccinators knew the national BCG vaccination schedule was at birth, MDVP became the main reason for their transferal of caregivers. That is, most of the caregivers were not able to get the BCG vaccine for their children immediately following delivery, as they were transferred out by nurses or by themselves (self-transferal) because vaccinators open a BCG vaccine only when they get enough children, as they strive to reduce BCG vaccine wastage.

*Just post-delivery on Wednesday at [Hospital], the nurses told me that I could either bring my baby on Thursday [BCG opening day] for vaccination or get the service from a nearby health centre. So, I preferred to come here [Health Centre] because it is close to my house. On the next day, my baby was vaccinated.* (Caregiver, female)

The vaccinators advised caregivers either to go immediately post-delivery discharge or on the 45th day after delivery. Differently, some other vaccinators preferred taking the phone numbers of caregivers and calling them when the required number of children attended.

*The nurses asked me which health facility is nearest to me, and I told them the name of the health centre. Then they gave me a piece of paper and told me that I could continue the follow-up on the 45th day after birth.* (Caregiver, female)

#### Blind transferal due to lack of formal communication and information

Besides the efforts of some vaccinators who knew the schedules of a few nearby health centres by calling nurses they knew on their mobile phones, most of them still had problems getting the exact dates and times when BCG and MCV vaccines could be opened at the other health facilities. As a result, they transferred caregivers without having clear information, so the caregivers had to pay unnecessary transport costs and felt that their time and energy were wasted.

*When a caregiver arrives on a date on which BCG and MCV are not given, we will either appoint the caregivers to another time or transfer them to another nearby health facility.* (Public health officer, male)

#### Absence of standardized tools and procedures in the transferal process

When some vaccinators transferred out caregivers, they advised them to take their child’s vaccination yellow card or provided transferal slips or a simple piece of paper, while others tried communicating with the facilities by phone before they transferred caregivers to them. Meanwhile, UHEWs used a medical referral form.

*We transfer caregivers out to the health facility where they need to go, and then we record that in our registration book. We do not record the name of the facility where the caregivers want to continue on the transfer paper.* (Vaccinator, female)

Two vaccinators described the challenge of providing vaccination services to those caregivers who visited them without any document, as they could not clearly tell which vaccines their children had already received. Some caregivers counted the eye ointment and vitamin K injection that were provided to their children during delivery as vaccines.

*Some caregivers come without any evidence and tell us, “He [their child] has been vaccinated”, but they do not know what vaccine he received. Sometimes, they come with cards that indicate his weight and tell us, “He received a vaccine in another facility, and that facility informed us to continue his vaccination here.” … Sometimes children come from hospitals with a small card that indicates SVD delivery. … Mothers who come from kebele do not bring anything for evidence, and they debate with us, they say, “He already received this,” or “He did not receive this.”* (Vaccinator, male)

#### Checking caregiver mobility

Most vaccinators asked caregivers for their residence addresses, whether they needed that day’s vaccination or would continue their follow-up at their health facility, and whether they had a plan to change their home or stay in the same house, and they also checked the appointment dates.

*When mothers come to our health centre, first we ask them their reason for coming here. If their intention is to get today’s vaccine, we don’t provide the service. We just send them back, but if they want to continue in our health centre due to change of residence or other reasons, we accept and vaccinate their children.* (Vaccinator, female)

### Feedback mechanisms

#### Absence of established formal feedback system

Most of the EPI focal persons noted that there was no system in place to follow the outcomes of transferred-out children. Only a few self-motivated vaccinators would call caregivers and ask them if they had gone to the receiver health facilities. Nonetheless, UHEWs had a chance to learn whether a transferred-out child had reached the health centre and received the required vaccine or not during their house-to-house visits.

*I think there are some gaps. We transfer out a lot of children, but there is no way for us to get feedback. But what we hope is that the caregivers will visit the health facilities because we inform them a lot about how beneficial the immunizations are for their babies.* (Vaccinator, female)

## Discussion

Linkages were first initiated between two units within a hospital or health centre – the delivery service unit and EPI unit – when a caregiver gave birth, as a newborn baby requires the first vaccine (BCG) at birth. Then, the linkages moved only among the EPI units of health facilities in the three-tier health system: between hospitals and hospitals, hospitals and health centres, health centres and health centres, and health centres and the community through UHEWs within or outside of the sub-city. Through the path of the linkages, the transferal process was established, and caregivers moved from initiating health facilities to receiving facilities, which were closer and more easily accessible to them. Almost all vaccinators transferred out caregivers who needed multi-dose vial (MDV) vaccines, particularly BCG and MCV.

Even though newborn vaccination (BCG, OPV-0, and hepatitis B) is a critical parameter for evaluating the overall performance of immunization programmes [10], most vaccinators transferred out caregivers without providing BCG in order “to minimize BCG wastage” – in adherence to the MDVP which permits certain vaccines to be stored for up to 28 days after opening, and any other actual or perceived thresholds for opening a vial [11]. As a result, they reported successful reduction of vaccine wastage rates as a strength; yet most caregivers wasted their time, energy, and money by travelling from one health facility to another. Similarly, caregivers faced the same challenges in getting MCV, as a change in the number of doses per container affects operational costs, timely coverage, safety, product costs, and policy [12].

In achieving maximum utilization of every dose, vaccinators need to be proactive with communication to ensure optimal attendance and timely vaccination of every child [13]; but even though communication activities are a crucial element of immunization programmes [14], none of the vaccinators knew the vaccination schedules of receiving health facilities through formal communication channels. Meanwhile, use of phone contacts to follow up is a feasible and cost-effective method for tracking children who have missed out [15] and has been used successfully by community health workers in some urban settings [16]. Despite some efforts to transfer caregivers using “transferal slips” or pieces of paper or through informal phone calls to vaccinators’ friends, unfortunately, there was no formally established system for transferring caregivers and obtaining feedback. Besides, there was no way for the receiver health facility to notify the first health facility from which the caregivers had been transferred about their arrival. Nonetheless, many caregivers became dissatisfied and did not continue follow-up due to travel to several health facilities and reappointments.

The main strengths of the study include the transferal processes observed among various types of health facilities with different characteristics. Although demand for vaccines and access to immunization services is a complex concept that is not external to supply systems [15, 17], the study covered caregivers from the demand side and UHEWs, vaccinators, medical directors, supervisors, EPI focal persons, and managers from the supply side, so the behaviour of both service providers and users was properly observed. Considering the more pervasive and common patterns of schedule non-completion among poorer urban mothers [18–21] and the identified gaps, the authors make the following appropriate recommendations: The Federal Ministry of Health (FMoH) should develop a protocol with standardized transferal tools to facilitate the transferal process and ensure accountability; support the appropriate implementation of MDVP; initiate a formal feedback provision system using SMS text messages; and build the capacity of vaccinators, EPI focal persons, supervisors, and managers to ensure formal transferal processes with feedback.

Potentially, the study’s findings have limited generalizability to other places because the linkages between health centres and health posts were not observed, as there are no health posts overall in the capital. Therefore, the linkage and transferal system between health centres and health posts should be investigated in the rural and pastoral regions of the country. On the other hand, it was not possible to get caregivers who travelled to places far outside the capital, so the sample might not be representative. Currently, there are emerging productions of new MDVs like PCV; user countries should conduct similar studies to assess the linkages and transferal processes in accessing BCG, measles, and PCV vaccines for children.

## Conclusion

Fragile linkages, transferal processes, the feedback system, and caregiver mobility affect follow-up visits for immunization services. Transferal of caregivers for the sake of minimizing MDV vaccine wastage without checking the vaccination schedules of receiving health facilities, using various types of tools and approaches, along with a poor follow-up and feedback system, cost caregivers extra time, energy, and money for transport to achieve timely services by moving from one health facility to another. Hence, the FMoH should ensure formal communication and transferal processes by developing guidelines, standardized tools, and procedures; initiating feedback systems using SMS text messages; and building vaccinator capacity.

## Data Availability

The data sets used and analysed during the current study are available from the corresponding author upon reasonable request.

## Abbreviations

BCG: Bacille Calmette–Guérin
EPI: Expanded programme of immunization
EPHI: Ethiopian Public Health Institute
FGD: Focus group discussion
FMoH: Federal Ministry of Health
IDI: In-depth interview
IPV: Inactivated poliovirus vaccine
KII: Key informant interview
OPV: Oral polio vaccine
MCV: Measles-containing vaccines
MRV: Measles-rubella vaccine
MDV: Multi-dose vial
MDVP: Multi-dose vial policy
PCV: Pneumococcal conjugate vaccine
PHCUs: Primary health care units
RV: Rotavirus vaccine
RI: Routine immunization
SMS: Short message service
SVD: Self vaginal delivery
UHEWs: Urban health extension workers
VPDs: Vaccine-preventable diseases

## Declarations

### Costs for publication

Publication costs are funded by WHO, Alliance for Health Policy and System Research (AHPSR), Decision-Maker Led Implementation Research (DELIR) initiative.
